# Structure challenges in the multivalency of Tau-microtubule interactions

**DOI:** 10.1002/cm.21788

**Published:** 2023-09-13

**Authors:** Eva Nogales, Elizabeth Kellogg

**Affiliations:** 1 Molecular and Cell Biology Department, University of California, Berkeley, CA, USA; 2 California Institute for Quantitative Biosciences (QB3), University of California, Berkeley, Berkeley, CA, USA; 3 Howard Hughes Medical Institute, University of California, Berkeley, Berkeley, CA, USA; 4 Molecular Biophysics and Integrative Bioimaging Division, Lawrence Berkeley National Laboratory, Berkeley, CA, USA; 5 Structural Biology Department, St. Jude Children’s Research Hospital, Memphis, TN

## Abstract

Structural studies aiming to visualize the interaction of Tau with microtubules face several challenges, the main concerning the fact that Tau has multiple microtubule-interacting regions. In particular, the four (or three) pseudo repeats of Tau bind to identical elements along the microtubule lattice but do it through non identical residues. Additionally, any given Tau molecule can use all its repeats or just one for its engagement with microtubules. Finally, the binding of one Tau is not necessarily in register with the respect to the next one. The mismatch in the microtubule and Tau repeats therefore challenge conventional modes of image analysis when visualizing these samples using cryo-electron microscopy. This commentary is dedicated to those challenges and ways to circumvent them while aiming for an atomic description of the Tau-tubulin interaction.

Microtubules (MTs) are ubiquitous cytoskeletal elements of eukaryotic cells that play a myriad of roles, all of them involving a number of microtubule-associated proteins (MAPs) that recognize, bind and often regulate MT activity. Polymers of repeating αβ-tubulin subunits, MTs are made of protofilaments that associate in a parallel, staggered manner to give rise to a pseudo-helical array of tubulin dimers. Within the MT lattice, lateral contacts are homotypic at all but one contact (perhaps more), involving α–α or β–β contacts, with one seam (perhaps more) in which the contacts are heterotypic (α–β and β–α), thus breaking helical symmetry. Structural studies of MTs and their interactions with MAPs have been the realm of cryo-EM (for some of our own reviews see (Nogales & Kellogg, 2017; Nogales & Zhang, 2016)), a technique that overcomes the requirement for crystallization, which is generally incompatible with polymer assembly. With the advent of technical improvements in electron microscopes, electron detectors and image processing software, the cryo-EM field has seen a burst of activity over the last decade that has also resulted in increase in the number of MT structures described during that period (for some of the latest and most spectacular results, you see these structural analyses of axonemes (Leung et al., 2023; Walton et al., 2023)).

Most of the in vitro studies of MAPs binding to MTs have involved one single MAP added to MT solutions in excess in order to obtain maximal occupancy of the binding site on the MT lattice. Such conditions take advantage of the tubulin repeat to obtain the structure of the MAP bound to it at the best possible resolution. Such saturation of the MT lattice by a single factor is obviously not representative of the in vivo situation in which many different factors may interact simultaneously with a given MT along its length, very likely at much lower stoichiometries, and perhaps affecting each other’s binding in the process. Still, in most cases the binding saturation used for cryo-EM studies is nonetheless totally compatible with the natural binding of the MAP of interest and an acceptable condition for the purpose of structural characterization. There are conditions, however, for which this compatibility breaks down. Think for example of the case of kinesin dimers. Under saturation conditions one of the motor heads will bind while the other, in a lower affinity state, will hover, unattached, unable to find a free site to bind to the MT. The solution when trying to visualize both heads engaged is to lower the concentration so that binding of the first head will occur at a site surrounded by open kinesin binding sites where the second head can land. This type of study has been carried, for example, by the Sindelar lab, which needed to use ingenious computational strategies to localize the double binding occurrences along the sparsely populated MT and obtained a structural description of those events (Liu et al., 2017). Another challenging situation is that of MAP7. First visualized bound to microtubules in the context of its interplay with Kinesin 1 on the MT surface (Ferro et al., 2022), it turned up to have a footprint on the MT larger than a tubulin dimer repeat (Adler et al., 2023). A challenging case that somewhat combines the issues just described for kinesin dimers and MAP7 is that of Tau.

In neurons, MTs interact with a family of “classical” MAPs that contribute to MT organization and stability and are critical to neuronal growth and function, including MAP-2, MAP-4, and tau. Tau, the molecular protagonist of this issue, localizes to axons and is the most abundant neuronal MAP. Intrinsically disordered, Tau from adult human neurons includes a central, MT-binding region of four pseudo-repeats (R1-4). Our lab was able to obtain the first atomic model of Tau binding to microtubules (Kellogg et al., 2018) and about a year after that of amyloid tau aggregates were first characterized at high-resolution also using cryo-EM (Fitzpatrick et al., 2017). In our study we assembled MTs in the presence of excess full-length tau, and obtained a cryo-EM reconstruction that showed tau as a narrow, discontinuous density following the outermost ridge on the MT surface along each protofilament, fully consistent with the low resolution cryo-EM reconstruction obtained years before by Al-Bassam and Milligan (Al-Bassam et al., 2002). Whether we examined full-length tau, or N- and C-terminally truncated tau constructs that included either all four repeats (4Repeat) or just the first two repeat sequences (2Repeat), the density corresponding to tau looked practically indistinguishable and corresponded to an extended chain of about 27 aa, with a connecting region of weaker density that would accommodate another 3–4 extended residues. Thus, each segment of the observed density could be attributed to a single repeat of 31 aa.

It is important to note that the cryo-EM structures for these tau constructs necessarily corresponded to an average of the different repeats engaging the MT surface. Several issues that are quite unique to tau as a multidomain MT binder need to be kept in mind, even when concentrating in binding of the pseudo-repeats (notice that there are other regions in tau that have also been implicated in MT binding (e.g.(Goode et al., 1997)), but they are not observed in cryo-EM structures, likely because they interact with the flexible C-terminal tails of tubulin in a highly dynamic manner (Hinrichs et al., 2012; McVicker et al., 2014)). First, the repeats are similar enough that at the resolutions attained (~4 Å) they will be practically impossible to distinguish, even if they had been properly aligned one with respect to the other. But that is most unlikely, because repeats would bind along different protofilaments out of register, that is, with no fixed relative position among them (see an example in Fig. 1 of a possible configuration of Tau molecules binding to the microtubule using different number of repeats, and with the repeats binding in a staggered manner from one protofilament to the other). Finding that register is like separating beads of identical size and different colors when we do not see color. Furthermore, under conditions of saturation, any particular Tau molecule likely would not engage using its 4 repeats, but likely via fewer, perhaps just one with the highest affinity (Fig. 1 shows Tau molecules that bind using, from right to left, 4, 3, 2, or just 1 repeat). As an ensemble, Tau molecules could be binding the microtubules with all possible repeat stoichiometries. The way we overcame these issues was to generate artificial Tau constructs that contained 4 identical copies of the same repeat (either four identical copies of R1 (R1×4) or of R2 (R2×4)). Maintaining 4 repeats helped binding by conserving the avidity allowed by multiple repeats but making them identical meant that averaging will give rise to a consistent structure corresponding to that of the used repeat. Using the densities obtained as restraints, we then utilized Rosetta to generate atomic models. The self-consistency of the structures for R1×4 and R2×4 (the solutions for R1 and R2 corresponded to the same sequence register) lent credibility to the atomic models and allowed us to propose a model of how R3 and R4 should also engage with the MT. In the context of full-length tau, repeats are likely to bind one after the other in tandem along a PF, promoting MT polymerization and stabilization by tethering tubulin dimers together across longitudinal interfaces This modular structure would explain how alternatively spliced variants (regulated developmentally or in disease states) can have essentially identical interactions with tubulin but different affinity based on the number of repeats present. But such model has yet to be corroborated by experimental data.

Cryo-EM studies are, in principle, the most direct way to provide a direct visualization of the complex engagement of tau’s four repeats but suffer from the shortcomings described above. As in the case of the kinesin dimer, any chance to visualize the “natural” engagement of tau with the MT without competition among the repeats is to allow binding at lower tau-tubulin stoichiometries. The challenge then is to identify the position of tau molecules and to align them to each other in the correct register (R1 with R1, R2 with R2, etc) to build signal and thus, resolution. This will require ingenuity to mark one particular position along the tau chain with a fiducial mark of enough electron density to be clearly visible, therefore allowing proper alignment of the tau chains at the single repeat level. Maybe more than one such fiducial will be required if we do not assume that all repeats are always engaged on the lattice. Obtaining a statistical description of the mode of engagement, which will be dynamic by nature, will itself be very useful for our understanding of Tau function. Finally, given the capacity of tau to self-interact giving rise to tau “condensates” of distinct biophysical properties (Siahaan et al., 2019; Tan et al., 2019), it will be important to see how being in or out of the regimen that allows condensate formation will affect the engagement of the tau repeats with tubulin, and vice versa. Ultimately, a single structural/biophysical technique will likely not be enough to provide a full view of Tau-MT interactions and multiple tools and approaches will need to be combined, included computation methods (Bhandare, Kumbhar, & Kunwar, 2019)Important challenges and exciting questions to be addressed in future structural studies of this intriguing MAP that just turned 50 years old for the scientific community (Weingarten et al., 1975).

## Figures and Tables

**Fig. 1 -:**
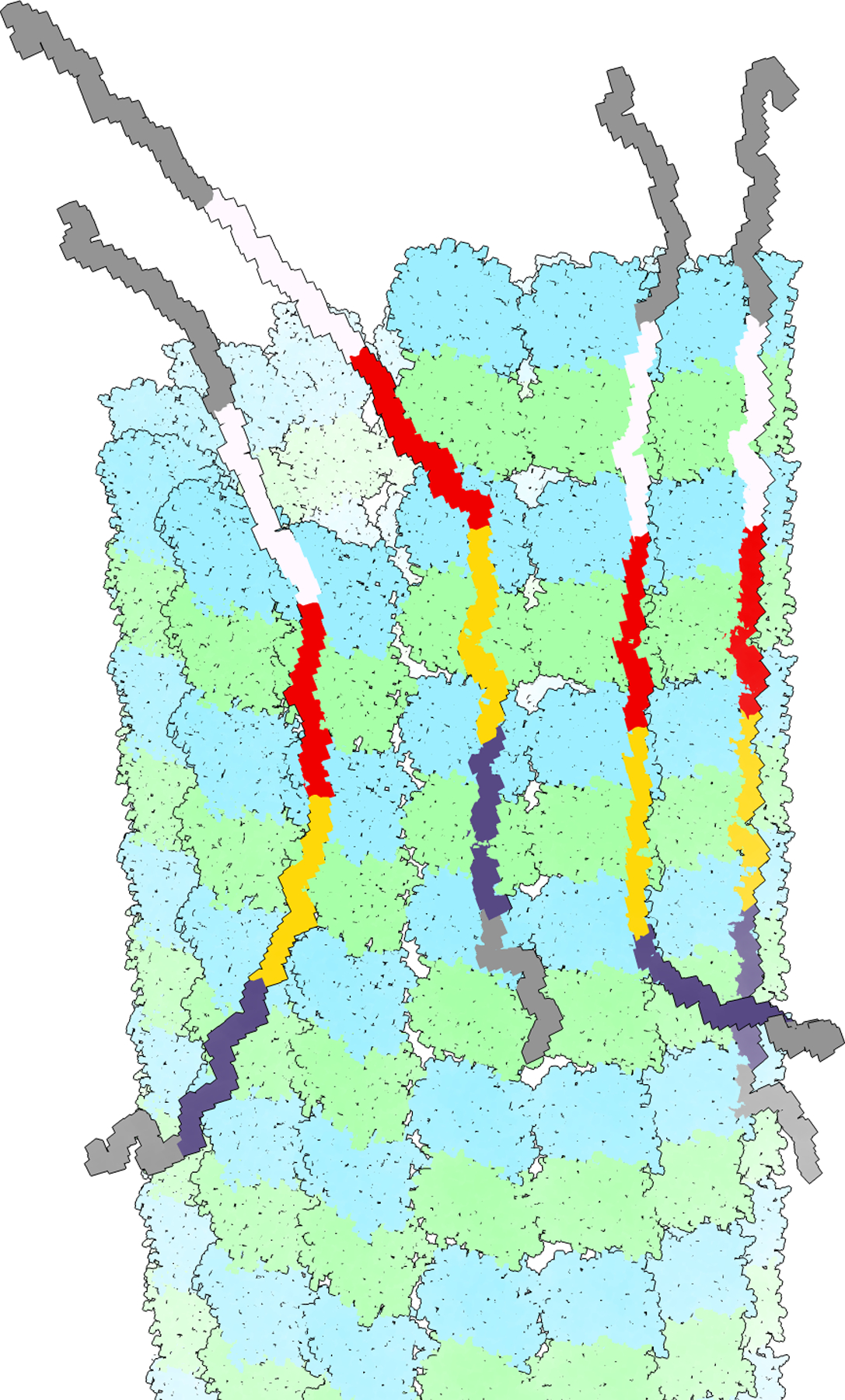
Schematic representation of possible binding modes of Tau on a microtubules. Each Tau molecule can engage all or a smaller number of its pseudo-repeats. Tau molecules bind along protofilaments with different registers, so that different repeats are bound to adjacent tubulin dimers from one protofilament to the next. Repeats R1 to R4 are shown in white, red, yellow and blue. Adjacent regions are shown in grey. From left to right the four tau molecules are engaged with the microtubule via 1, 2, 3 or all 4 repeats.
